# The Role of Tyrosine Kinases as a Critical Prognostic Parameter and Its Targeted Therapies in Ewing Sarcoma

**DOI:** 10.3389/fcell.2020.00613

**Published:** 2020-07-09

**Authors:** Wook Jin

**Affiliations:** Laboratory of Molecular Disease and Cell Regulation, Department of Biochemistry, School of Medicine, Gachon University, Incheon, South Korea

**Keywords:** Ewing sarcoma, tyrosine kinases, aggressiveness, therapeutic target, chemoresistance, clinical pathogenesis, acquisition of self-renewal traits

## Abstract

Ewing sarcoma (ES) is a rare, highly aggressive, bone, or soft tissue-associated tumor. Although this sarcoma often responds well to initial chemotherapy, 40% of the patients develop a lethal recurrence of the disease, with death recorded in 75–80% of patients with metastatic ES within 5 years, despite receiving high-dose chemotherapy. ES is genetically well-characterized, as indicated by the EWS-FLI1 fusion protein encoded as a result of chromosomal translocation in 80–90% of patients with ES, as well as in ES-related cancer cell lines. Recently, tyrosine kinases have been identified in the pathogenesis of ES. These tyrosine kinases, acting as oncoproteins, are associated with the clinical pathogenesis, metastasis, acquisition of self-renewal traits, and chemoresistance of ES, through the activation of various intracellular signaling pathways. This review describes the recent progress related to cellular and molecular functional roles of tyrosine kinases in the progression of ES.

## Introduction

The tyrosine kinase family, including both receptor tyrosine kinases (RTK) and non-receptor tyrosine kinases, triggers a cascade of downstream signaling pathways that control the complex biological process of cells, including proliferation, cellular organization, and differentiation. In normal cells, RTK activity is induced by the tightly regulated interaction between receptor-ligands, receptor-proteins, and reduced by negative regulation of protein tyrosine phosphatases (Zhang et al., 2009; Casaletto and McClatchey, 2012; Du and Lovly, 2018). For example, the tropomyosin-related kinase A (TrkA) receptor, which is activated via its ligand, nerve growth factor (NGF)-induced dimerization, contributes to the neuronal differentiation (Shen and Maruyama, 2011). Also, Platelet-derived growth factor (PDGF)-β-mediated activation of platelet-derived growth factor receptor (PDGFR) can activate intercellular pathways through complex formation between PDGFR and Src family tyrosine kinases (Kypta et al., 1990). Moreover, the density-enhanced phosphatase 1 (DEP1) blocks the internalization of vascular endothelial growth factor receptor (VEGFR) 2 by inducing dephosphorylation of VEGFR 2 (Lampugnani et al., 2003). However, dysregulation of RTKs (as identified in various human cancers) may result in their activation by ligand-dependent or independent autophosphorylation, gain-of-function mutations, and gene amplification-mediated overexpression (Zhang et al., 2009; Casaletto and McClatchey, 2012; Du and Lovly, 2018). RTK activation promotes cell proliferation, increases cell migration, motility, and disseminating ability, and confers drug resistance (Shibue et al., 2017; Jiao et al., 2018; Dongre and Weinberg, 2019). Hence, a variety of RTKs have been identified as therapeutic targets for anticancer drug development.

Notably, activation and overexpression of RTKs have been observed in Ewing sarcoma (ES), suggesting that tyrosine kinases may play key roles in the pathogenesis of ES. Clinical or preclinical inhibitors of tyrosine kinases have been developed and tested as molecular targeting therapy in ES (Gaspar et al., 2015; Grunewald et al., 2018). Herein, we explore recent advances in our understanding of the effects of tyrosine kinase activation in ES, particularly focusing on mechanisms by which tyrosine kinases are linked to the progression of ES. Determining molecular mechanisms through which tyrosine kinases influence the pathogenesis of ES may offer a novel strategy to treat ES, as well as to increase the efficacy of current therapeutics.

## Ewing Sarcoma

Although ES has a low incidence (accounting for less than 10% of human malignancies), it is the second most common round cell malignancy occurring in the primary soft tissues and bones of children, adolescents, and young adults, with 12% of pediatric malignancies (Grier, 1997). Additionally, ES has been detected in the ribs, spine, skull, and bones of hands, arms, legs, feet, and pelvis (Grunewald et al., 2018). Overall, 75% of ES patients with a localized tumor have demonstrated improved survival through a combination of multi-agent cytotoxic chemotherapy and local-control measures (surgical resection and radiotherapy) (Balamuth and Womer, 2010). Notably, the 5-year overall survival (OS) in ES patients is 87.5% at 10 years and 79.2% at 20 years, with patient mortality reported as 68.9% (Davenport et al., 2016). Approximately 20–25% of ES patients with local and distant treatment failure developed distant metastasis after completion of systemic and local treatment (Nesbit et al., 1990; Gaspar et al., 2015).

In another study, 30%–40% of ES patients presented local and distant recurrence, closely associated with poor prognosis. The 5-year survival rate of patients with metastases significantly decreases to less than 30%, and the most common recurrent sites include the bone (56.5%), lung (52.2%), brain (6.5%), and bone marrow (6.5%) (Rodriguez-Galindo et al., 2002; Riggi and Stamenkovic, 2007; Gaspar et al., 2015). Furthermore, chemotherapy and radiotherapy can induce secondary malignant neoplasms, and these secondary malignancies worsen the survival rates in ES patients (Longhi et al., 2012). In ES patients who underwent radiotherapy, local recurrence was observed in 30–40% of patients (Zucman et al., 1993; DuBois et al., 2015), after which the survival rate was significantly reduced to less than 20–25% (Ahrens et al., 1999; Ross et al., 2013).

A specific chromosomal translocation *t*(11;22) (q24;q12) occurs in ES (Delattre et al., 1992), and this chromosomal translocation results in the generation of a chimeric EWS-FLI1 fusion protein, created by the fusion of the amino-terminal portion of the FET family gene *EWSR1* with the carboxyl-terminal DNA-binding domain of the ETS family gene, *FLI1*. Approximately 80–90% of patients with ES harbor the EWS-FLI1 fusion protein. This protein, as an aberrant transcription factor, is reportedly involved in the progression of ES (Delattre et al., 1992; Bonin et al., 1993; May et al., 1993). In the molecular diagnosis of ES, one key feature is the detection of translocation involving the *EWSR1* gene on chromosome 22 band q12 by fluorescent *in situ* hybridization (FISH)-based detection or using the RT-PCR method (Grunewald et al., 2018). Additionally, the fusion of EWSR1 to the DNA-binding domain of ERG results in the EWS-ERG protein, which demonstrates functions similar to EWS-FLI1 (Sorensen et al., 1994), and EWS-ERG has been identified in 12.3% of ES patients (Delattre et al., 1994). EWS-ERG was detected in circulating tumor cells of ES patients with large tumors and has been correlated with reduced survival in these patients (Schleiermacher et al., 2003).

ES patients demonstrate a chromosomal abnormality as a prognostic indicator. In total, 77.6% (38/49), 26.5% (13/49), 26.5% (13/49), 26.5% (13/49), and 24.5% (12/49) of ES patients contained trisomy 8, 2, 5, 12, and 20, respectively. Specifically, trisomy 20 was closely associated with a worsened OS (Roberts et al., 2008). Moreover, ES induces the upregulation of the CD99 protein and caveolin 1 (CAV1), as diagnostic markers. CD 99 is a single-chain type-1 membrane glycoprotein, highly expressed in 90–97% of ES patients (Ambros et al., 1991; Riggi and Stamenkovic, 2007; Llombart-Bosch et al., 2009). CAV1, another diagnostic immunomarker, is highly expressed in 96% of ES patients, and its upregulation is significantly associated with CD99 expression. Additionally, CAV1 is detected in CD99-negative ES patients (Llombart-Bosch et al., 2009).

For the successful treatment of ES patients, most protocols of multi-agent cytotoxic chemotherapy involve vincristine/ifosfamide/doxorubicin/etoposide (VIDE) administration (Juergens et al., 2006). Furthermore, alternative multidrug chemotherapy protocols contain cyclophosphamide, topotecan, and etoposide. In standard-risk patients, the administration of vincristine/dactinomycin/ifosfamide/doxorubicin (VAIA) therapy presented no differences in the event-free survival (EFS) and OS hazard ratio when compared with VACA therapy (cyclophosphamide replacing ifosfamide). However, cyclophosphamide revealed a higher incidence of hematological toxicity. In high-risk patients who received chemotherapy including etoposide, the EFS and OS hazard ratio demonstrated a 17% and 15% reduction in the risk of an event or death relative to VAIA therapy, respectively (Paulussen et al., 2008). Moreover, the addition of vincristine/topotecan/cyclophosphamide (VTc) to the standard five-drug chemotherapy (vincristine/doxorubicin/cyclophosphamide/ifosfamide/etoposide [VDCIE]) presented better clinical benefits for ES patients. The addition of VTc to standard therapy demonstrated no toxicities, and in patients with ES, the 5-year OS and EFS were 88% and 79.5% when compared with standard 3-week cycles, respectively (Mascarenhas et al., 2016).

## The Functional Role of Receptor Tyrosine Kinases in the Progression of ES and Its Inhibitors

### Insulin-Like Growth Factor I Receptor (IGF-1R) and Its Inhibitors

**FIGURE 1:**
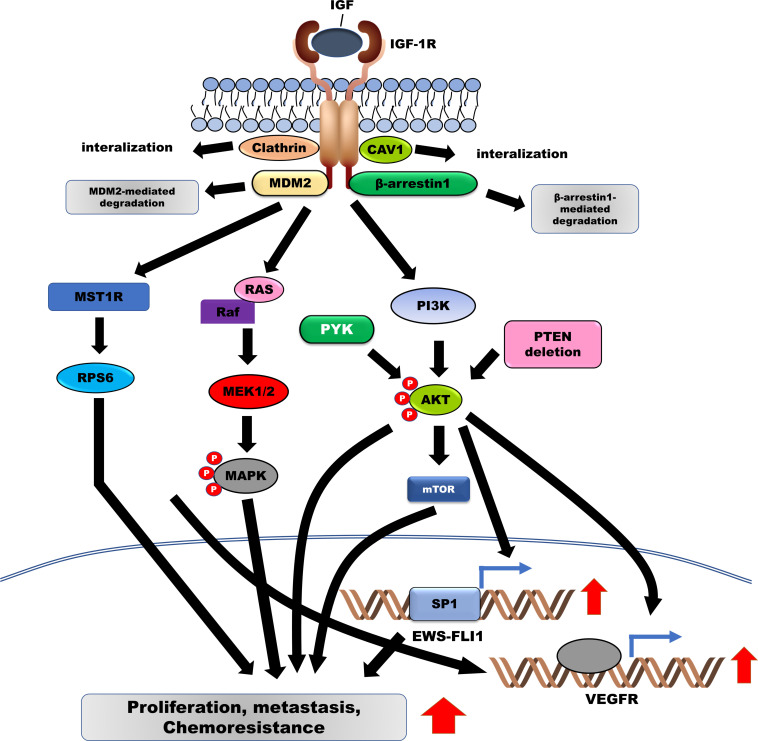
Induced activation of IGF-1R facilitates the survival, metastasis, and chemoresistance in Ewing sarcoma (ES) by activating downstream signaling pathways. IGF/IGF-1R enhances the pathogenesis of ES by upregulating EWS-FLI-1 and VEGFR via the activation of the RAS/MAPK/ERK and PI3K/AKT signaling pathways. Additionally, IGF-1R is degraded by MDM2 and β-arrestin-1. IGF-1R, Insulin-like growth factor 1 receptor; VEGFR, vascular endothelial growth factor; MDM2, murine double minute-2.

Insulin-like growth factor I receptor-1 mediated IGF-1R activation induces proliferation, epithelial-mesenchymal transition (EMT), metastasis, drug resistance, and tumor recurrence (Li et al., 2017). The promoter activity of IGF-1R is significantly activated by the binding of EWS-WT1 with the -331 to -40 region of the IGF-1R promoter in desmoplastic small round cell tumor (DSRCT), a malignant soft tissue sarcoma occurring in young children (Karnieli et al., 1996). This indicates that IGF-1R may promote the transcriptional expression of EWS fusion genes by inducing distinct cellular pathways involved in the pathogenesis of various types of cancer. In one study, IGF-1R was reportedly upregulated in all the tumor samples, including those from ES and synovial sarcoma patients, and inhibition of the IGF-1R signaling pathway resulted in a loss of the invasive ability of ES cells (Figure 1; Scotlandi et al., 1996; Xie et al., 1999; Asmane et al., 2012). Another report revealed that IGF-1R was upregulated in 93% of ES patients (Mora et al., 2012; Table 1). Additionally, IGF-1R activation is required for the EWS-1/FLI1-mediated transformation of ES cells (Toretsky et al., 1997). The sub-cellular localization of IGF-1R is associated with the poor survival observed in ES patients. Furthermore, nuclear localization of IGF-1R markedly increases prolonged progression-free survival (PFS) and OS in ES patients when compared with the cytoplasmic localization of IGF-1R (Asmane et al., 2012; Table 1).

**TABLE 1:** Involvement of tyrosine kinases in Ewing sarcoma.

**Tyrosine kinase**	**Status**	**Methods**	**Detection**	**References**
Insulin-like growth factor 1 receptor (IGF-1R)	Overexpressed Overexpressed	IHC IHC	16/16 (60 − 100%) 28/30 (93%)	Asmane et al., 2012; Mora et al., 2012
Fibroblast growth factor receptors (FGFRs)	Overexpressed Overexpressed	IHC/qRT-CR, IHC	7/9 (77.8%) 61/136 (44.85%)	Kamura et al., 2010; Agelopoulos et al., 2015
Eph receptor A2 (EphA2)	Overexpressed	IHC	66/73 (90.4%)	Garcia-Monclus et al., 2018
c-kit stem cell receptor (CD117)	Overexpressed Overexpressed Overexpressed	IHC IHC IHC	11/17 (65%) 27/71 (38%) 22/31 (71%)	Smithey et al., 2002; Ahmed et al., 2004; Do et al., 2007
Human epidermal growth factor receptor 2 (HER2) Human epidermal growth factor receptor 2 (HER4)	Overexpressed Overexpressed	IHC IHC	18/113 (32%) 45/78 (57.7%)	Scotlandi et al., 2005; Mendoza-Naranjo et al., 2013
MET anaplastic lymphoma kinase (ALK)	Overexpressed Overexpressed	IHC IHC	43/50 (86%) 41/59 (70%)	Fleuren et al., 2013
Platelet-derived growth factors receptor (PDGFR)β Platelet-derived growth factors receptor (PDGFR)α Platelet-derived growth factors receptor (PDGFR)β	Overexpressed Overexpressed Overexpressed	IHC qRT-PCR, IHC qRT-PCR, IHC	47/52 (90.4%) 10/19 (52%) 15/19 (79%)	Uren et al., 2003; Wang et al., 2009
Protein tyrosine *kinase* (PTK) *Lyn*	Overexpressed	WB	16/15 (86.7%)	Guan et al., 2008
FMS like tyrosine kinase 3 (*FLT3; CD135*)	Overexpressed	RT-PCR	17/19 (89.4%)	Timeus et al., 2001
AXL receptor tyrosine kinase	Overexpressed	RT-PCR, IHC	20/29 (69%)	Fleuren et al., 2014
Spleen tyrosine kinase (SYK)	Activated	IHC	14/35 (40%)	Sun et al., 2017

*IHC, Immunohistochemistry; qRT-PCR, Quantitative Real-time polymerase chain reaction; WB, Western blotting; RT-PCR, Real-time polymerase chain reaction.*

Importantly, activation of IGF-1R signaling prevents the apoptosis of ES cells induced by the cytotoxic activities of anticancer drugs, such as doxorubicin, by activating phosphoinositide-3-OH kinase/AKT pathway (Toretsky et al., 1999) and enhancing the transformation of mouse fibroblasts through transcriptional induction of EWS-FLI1 via the PI3K/AKT-mediated upregulation of specificity protein 1 (SP1) transcription factor (Toretsky et al., 1997; Giorgi et al., 2015). Introduction of antisense IGF-1R decreased cell proliferation and tumor growth of ES cells *in vivo* and *in vitro* (Scotlandi et al., 2002b). Both *in vivo* and *in vitro*, the expression of the IGF-I receptor dominant-negative mutant in ES cells markedly reduced the growth rate, tumor formation, metastases, and chemoresistance against doxorubicin (Scotlandi et al., 2002a). Activation of IGF-1R by IGF-1 activates AKT and extracellular-signal-regulated kinases (ERK) 1/2 in ES cells. However, inhibition of RAS/MEK/MAPK activation failed to rescue the proliferation of ES cells induced by IGF-1 treatment, indicating that compensatory activation of the RAS/MEK/MAPK signaling pathway may inhibit only IGF-1R (Benini et al., 2004). Moreover, investigation of 56 ES patients receiving neoadjuvant chemotherapy for prognostic and therapeutic purposes demonstrated positive correlations between the levels of IGF-1R and phospho-ERK (*ρ* = 0.286, *p* = 0.031, respectively), IGF-1R and phospho-AKT (*ρ* = 0.269, *p* = 0.045, respectively), phospho-AKT and phospho-ERK (*ρ* = 0.460, *p* = 0.000, respectively), and phospho-ERK and phospho-mTOR (mammalian target of rapamycin) (*ρ* = 0.273, *p* = 0.038, respectively) (van de Luijtgaarden et al., 2013). Furthermore, activation of IGF-1R and mTOR and expression of EWS-FLI1 were strongly correlated with high BMI-1 levels, which is overexpressed in 80% of ES patients and induces tumor growth in a xenograft model (van Doorninck et al., 2009).

Clinical and preclinical IGF-1R inhibitors are being utilized as monotherapy or in combination with anti-neoplastic inhibitors to inhibit ES (Table 2). Internalization and ubiquitination of IGF-1R are required for enhancing the sensitivity of the IGF-1R inhibitor. Both clathrin- and caveolin-1 (CAV-1)–dependent endocytosis-mediated internalization of IGF-1R are localized in the cytoplasm via complex formation between IGF-1R and both clathrin and CAV-1. Inhibition of clathrin- and CAV-1-dependent IGF-1R internalization following treatment with endocytosis inhibitors such as chlorpromazine (CPMZ) and methyl-β-cyclodextrin (MCD) rendered ES cells more sensitive to NVP-AEW541, an IGF-1R inhibitor (Martins et al., 2011). PU-71, heat shock protein (HSP) 90 inhibitor, is currently under evaluation for patients with solid metastatic tumors, lymphoma, and myeloma (ClinicalTrials.gov identifier: NCT01393509, NCT03166085, and NCT01269593). PU-71 treatment markedly suppressed the survival and metastatic ability of ES cells through inhibition of IGF-1R, AKT, and pERK activation, as well as downregulation of RAF-1, c-MYC, c-KIT, IGF1R, hTERT, and EWS-FLI1 expression. Furthermore, the combination of PU-71 and bortezomib, as a proteasome inhibitor, significantly inhibited tumor growth and metastasis in ES xenograft models (Ambati et al., 2014).

**TABLE 2:** Efficacy of Insulin-like growth factor 1 receptor (IGF-1R) inhibitors in combination with other inhibitors in treating Ewing sarcoma.

**Target of inhibitors**	**Inhibitor/Clinical trial identifier**	**Drug Type**	**Phase**	**Efficacy of Drug**	**References**
IGF-1R	Teprotumumab/NCT00642941	mAb	Phase 2	1% of patients had SD, and 8.7% of patients achieved PR	Pappo et al., 2011
IGF-1R	Figitumumab/NCT00560235	mAb	Phase 1/2	23% of patients had SD and 14.2% of patients achieved PC	Juergens et al., 2011
IGF-1R + mTOR	IMC-A12 + Temsirolimus/NCT00678769	mAb + Chem	Phase 1	50% of patients showed no disease but exhibited both mTOR and RAS/Raf/ERK activation	Subbiah et al., 2011
IGF-1R	BMS-536924	Chem	Cell line	This treatment induced sensitivity against ES cells by suppressing activation of IGF-1R and RPS6	Potratz et al., 2010
EWS-FLI1 + IGF-1R	Trabectedin + OSI-906	Chem	Cell line/Xg	This treatment synergistically increased cytotoxic effects in ES cells	Amaral et al., 2015
IGF-1R + CDK4/6	OSI-906 + Palbociclib	Chem	Xg	This combination markedly decreased tumor growth	Murakami et al., 2016
IGF-1R	Picropodophyllin/NCT01721577	Chem	Phase 1	This treatment inhibited cell survival by suppression of IGF-1R-mediated AKT activation	Aiken et al., 2017
IGF-1R + mTOR	Cixutumumab + Temsirolimus/NCT01614795	Chem	Phase 2	31% of patients achieved SD for more than 5 months, and 29% of patients had more than 20% of tumor regression	Naing et al., 2012
IGF-1R	Ganitumab/NCT00563680	mAb	Phase 2	49% of the ORR demonstrated SD with a good response, and 6% of the ORR exhibited PR	Tap et al., 2012
IGF-1R + CDK4/6	Ganitumab + Palbociclib/NCT04129151	mAb + Chem	Phase 2	This therapeutic strategy is currently under evaluation	UE
IGF-1R	Metformin	Chem	Cell line/Xg	Metformin significantly induced cell death of ES cells	Nangia-Makker et al., 2014
IGF-1R	allogeneic NK cell infusion and adoptive T-cell therapy/NCT02409576	IT	Phase 1/2	NK cell immunotherapy is now under evaluation, and IGF-1R-CAR-T and ROR1-CAT-T cells derived from sarcoma patients significantly increased the cytotoxicity of ES cells and reduced tumor growth	Jamitzky et al., 2015

*IGF-1R, Insulin-like growth factor 1 receptor; mAb, monoclonal antibody; Chem, Chemical; SD, Stable disease; PR, Partial response; CR, Complete recovery; ORR, Objective recovery rate; UE, Under Evaluation; Xg, Xenograft; IT, Immunotherapy.*

In patients with ES, IGF-1R inhibitors in combination with mTOR inhibitors could enhance the clinical benefits of therapy. The deletion of phosphatase and tensin homolog on chromosome 10 (PTEN) was identified in 25% of the ES patients, consistent with the *PTEN* deletion in ES cells, which eventually resulted in the reduced sensitivity to IGF-1R inhibitors, including NVP-AEW541 and OSI-906, and activation of the AKT pathway in the ES cells. Interestingly, the loss of PTEN expression in ES cells potentiated the autophagic response by increasing responsiveness to temsirolimus, a mammalian target of rapamycin (mTOR) inhibitor (Patel et al., 2014; Niemeyer et al., 2015).

In ES cells, constitutive activation of IGF-1R and its downstream PI3K/AKT/mTOR pathway results in the resistance to bromodomain and extra-terminal domain (BET) inhibitors. However, combining IGF-1R inhibitors with BET inhibitors, or mTOR inhibitors, presents a synergistic effect on the inhibition of survival and tumor growth of ES cells both *in vivo* and *in vitro* (Loganathan et al., 2019). Additionally, IGF-1R inhibitor-resistance in ES cells was closely correlated with the activation of IRS1, PI3K, signal transducer and activator of transcription (STAT)-3, Src, and p38 MAPK. Both IGF-1R inhibitor and mTOR inhibitor treatment significantly reduced the tumor growth in ES xenografts through inhibition of IGF-1R/PI3K/AKT/mTOR, and other signaling pathways, including MEK1/2, JAK/STAT3, TGF-β, and G-protein coupled receptors (Lamhamedi-Cherradi et al., 2016). Treatment with BMS-536924, an IGF-1R inhibitor, induced cell death, and cell cycle arrest in ES cells by suppressing activation of IGF-1R and RPS6. However, activation of MST1R, which is highly expressed in 50% of ES patients, blocked the BMS-536924-mediated cell death of ES cells by activating RPS6, a distal IGF1R effector. Treatment with rapamycin, a mTOR1 inhibitor, markedly reduced RPS6 activation in BMS-536924-resistant ES cells, inducing cell death in BMS-536924-resistant ES cells (Potratz et al., 2010). A combination of IMC-A12 (an IGF-1R) and rapamycin presented an enhanced therapeutic effect, demonstrating a prolonged response when compared with IMC-A12 treatment alone (Kolb et al., 2012). Moreover, patients who acquired resistance to a combination of IMC-A12 and temsirolimus (an mTOR inhibitor) (ClinicalTrials.gov identifier: NCT00678769) revealed activation of mTOR, STAT3, and AKT pathways after receiving this combination treatment (Subbiah et al., 2011).

Treatment with TAE226, a dual inhibitor of IGF-1R and focal adhesion kinase (FAK), significantly suppressed proliferation and tumor growth of ES cells *in vivo* and *in vitro* by inducing apoptosis by inhibiting IGF-1R, FAK, and AKT activation when compared with PF-562,271, a dual inhibitor of FAK and proline-rich tyrosine kinase 2 (Pyk2). TAE226 inhibited the activation of the brain-derived neurotrophic factor (BDNF), epidermal growth factor receptor (EGFR), and transforming growth factor (TGF) -β signaling pathways, regulated by IGF-1R and FAK (Moritake et al., 2019). Loss of CDK4 as an ES-selective dependency gene markedly reduced the growth of ES cells, but IGF-1R overexpression promoted the acquisition of resistance against the cyclin-dependent kinase (CDK) 4/6 inhibitor. Combination treatment with an IGF-1R inhibitor and CDK4/6 inhibitor demonstrated synergistic activity by inhibiting tumor growth and survival of ES cells *in vivo* and *in vitro* through the suppression of PKC/AKT/mTOR and activation of RB (Guenther et al., 2019). Furthermore, AZD3463, as a dual inhibitor of anaplastic lymphoma kinase (ALK) and IGF-1R, in combination with vorinostat, a histone deacetylase inhibitor, and temozolomide, an alkylating agent, induce cell death in ES cells by significantly inhibiting the activation of STAT3/AKT activation (Sampson et al., 2015).

N-linked glycosylation of IGF-1R is essential for its translocation to the cell surface. Treatment of IGF-1R with glycosylation inhibitors, including tunicamycin and the 3-hydroxy-3-methylglutaryl coenzyme A (HMG-CoA) reductase inhibitor, lovastatin, reduced the activation and expression of IGF-1R, subsequently reducing the survival of ES cells (Girnita et al., 2000b). Treatment with RITA (reactivating p53 and inducing tumor apoptosis), a furanic compound, significantly suppressed cell growth and metastatic potential in ES cells via RITA-induced downregulation of IGF-1R as a transcriptional target of p53, as well as the degradation of IGF-1R by RITA-mediated upregulation of murine double minute-2 (MDM2) (Di Conza et al., 2012).

Treatment with teprotumumab (R1507), an anti-IGF-1R antibody, completely inhibited the growth of ES cells by suppressing activation of the IGF-1R/IRS-1/AKT axis (Huang et al., 2011). Following this treatment, the complete response rate was 10% of the objective response rate (ORR), and the median OS was 7.6 months, indicating that teprotumumab treatment is well-tolerated. Teprotumumab has an extremely safe toxicity profile in patients with recurrent or refractory ES (ClinicalTrials.gov identifier: NCT00642941) (Pappo et al., 2011); however, it was reportedly inactive in patients presenting other types of sarcoma (Pappo et al., 2014). In January 2020, the FDA-approved teprotumumab as the first drug for the treatment of patients with thyroid eye disease (U.S.F.A.D. Administration, 2020a).

Figitumumab, a monoclonal anti-IGF-1R antibody, significantly reduces cell proliferation of ES cells by suppressing signaling pathways downstream to IGF-1R, including MAPK/ERK and PI3K/AKT pathways (Zheng et al., 2012). Additionally, figitumumab treatment increased β-arrestin1 expression, which inhibited the survival of ES cells by decreasing IGF-1R expression via β-arrestin1-mediated ubiquitination, following the formation of the IGF-1R/β-arrestin1 complex (Zheng et al., 2012). Figitumumab was evaluated in ES patients with metastases in phase 1/2 clinical trials. Reportedly, the complete response rate (disease stabilization) and partial response rate were 23% and 14.2% of the ORR, respectively. Additionally, in a phase 2 trial, figitumumab showed prolonged OS in ES patients (ClinicalTrials.gov identifier: NCT00560235) (Juergens et al., 2011).

Furthermore, human monoclonal antibodies have been developed and evaluated for the treatment of solid tumors, including ES. As cixutumumab (an IGF-1R inhibitor) monotherapy demonstrated limited single-agent activity in a phase 2 trial (ClinicalTrials.gov identifier: NCT00831844) in ES patients, a combination of cixutumumab and temsirolimus was used, revealing that 31% of patients achieved stable disease (SD) for more than 5 months and 29% of patients demonstrated more than 20% tumor regression (Naing et al., 2012). A phase 2 clinical trial of ganitumab (ClinicalTrials.gov identifier: NCT00563680), a monoclonal anti-IGF-1R antibody, revealed its well-tolerated and safe profile. In the trial, patients with a 49% ORR demonstrated SD with a good response, while those with 6% ORR exhibited partial response (Tap et al., 2012). However, IGF-1R-directed monotherapies, including figitumumab, cixutumumab, teprotumumab, ganitumumab, or cixutumumab, in combination with temsirolimus, presented only modest efficacy in ES patients (van Maldegem et al., 2016).

Currently, ganitumab monotherapy and a combination of palbociclib and ganitumab are under evaluation in phase 3 clinical trial as add-on therapy in patients with metastatic ES (ClinicalTrials.gov identifier: NCT02306161 and NCT04129151). Although anti-IGF-1R antibodies have been tested in clinical trials, these antibodies remain unapproved by the FDA.

Trabectedin (ET-743), an FDA-approved drug for the treatment of unresectable or metastatic liposarcoma or leiomyosarcoma (ClinicalTrials.gov identifier: ET743-SAR-3007 and NCT01343277) (Gordon et al., 2016; Barone et al., 2017), demonstrates antitumor activity against ES cells by inhibiting the binding of EWS-FLI1 to the promoter of its target genes, *TGF*β*R2* and *CD99.* Additionally, trabectedin in combination with OSI-906 (linsitinib), an IGF-1R inhibitor, markedly increases cytotoxic effects in ES cells by synergistically interacting with the anti-IGF-1R antibody or trabectedin (Amaral et al., 2015). Notably, ES patients with a genetic alteration owing to the *CDKN2A/B* loss and a *FUS-ERG* fusion-derived orthotopic xenograft (PDOX) model exhibited a similar histomorphological appearance. A combination of OSI-906 and Palbociclib (a CDK4/6 inhibitor) markedly decreased tumor growth in a PDOX model by suppressing the CDK4/6 and IGF-1R pathways (Murakami et al., 2016).

In a phase − clinical trial for picropodophyllin (AXL1717), a novel IGF-1R inhibitor, performed in patients with relapsed malignant astrocytomas, 44% of patients demonstrated prolonged SD for 12 months (ClinicalTrials.gov identifier: NCT01721577) (Aiken et al., 2017). Picropodophyllin inhibits cell survival in ES cells by suppressing IGF-1R-mediated AKT activation (Wu et al., 2015).

Metformin (1,1-dimethyl biguanide hydrochloride) is an FDA-approved drug indicated for the treatment of type 2 diabetes, reducing glucose concentration by inhibiting mitochondrial respiration and mitochondrial glycerophosphate dehydrogenase (Foretz et al., 2019). Recently, metformin was assessed in combination with chemotherapeutic drugs and it reportedly inhibited the mTOR pathway via suppression of IGF-1R activation, by decreasing the levels of insulin-like growth factor (IGF) (Engelman and Cantley, 2010). Metformin, in combination with 5-fluorouracil and oxaliplatin (FuOX), induced cell death in chemoresistant colon cancer cells by suppressing cancer stem cells (CSC) characteristics, STAT3, and NF-κB activation (Nangia-Makker et al., 2014) and suppressing STAT3 and NF-κB activation (Lin et al., 2013; Feng et al., 2014; Yue et al., 2015; Esparza-Lopez et al., 2019). ES demonstrates high metabolic and glycolytic activity, and a combination of 2-deoxy-D-glucose (2DG) with metformin induces cell death in ES via metformin-induced inhibition of mitochondrial respiration and 2DG-induced inhibition of aerobic glycolysis (Dasgupta et al., 2017). Furthermore, a combination of metformin and imatinib reduced the proliferation of ES cells by inducing cell cycle arrest and inhibiting tumor growth and metastasis in mice (Nan et al., 2020).

Natural killer (NK) cells and chimeric antigen receptor (CAR)-based immunotherapy for adoptive cellular immunotherapy have been applied for the effective treatment of ES, inducing the activation and expansion of NK cells by inhibiting IGF-1R expression, without affecting the immunophenotypes of NK cells and their degranulation response to ES cells, and subsequently maintaining potent antitumor effects against ES cells (Jamitzky et al., 2015). As NK cell therapy has reported safety and efficacy, as well as good tolerance, in leukemia patients, clinical trials of allogeneic NK cell infusions in patients with metastatic ES and high-risk rhabdomyosarcoma (RMS) are currently ongoing (ClinicalTrials.gov identifier: NCT02409576). IGF-1R and tyrosine kinase-like orphan receptor 1 (ROR1) are highly expressed in ES cells, and the application of adoptive T-cell therapy using IGF-1R-CAR-T and ROR1-CAT-T cells derived from sarcoma patients significantly increases ES cell death and reduces tumor growth of sarcoma xenografts (Huang et al., 2015).

### Fibroblast Growth Factor Receptors (FGFRs)/Their Ligand (FGF) and Its Inhibitors

**FIGURE 2:**
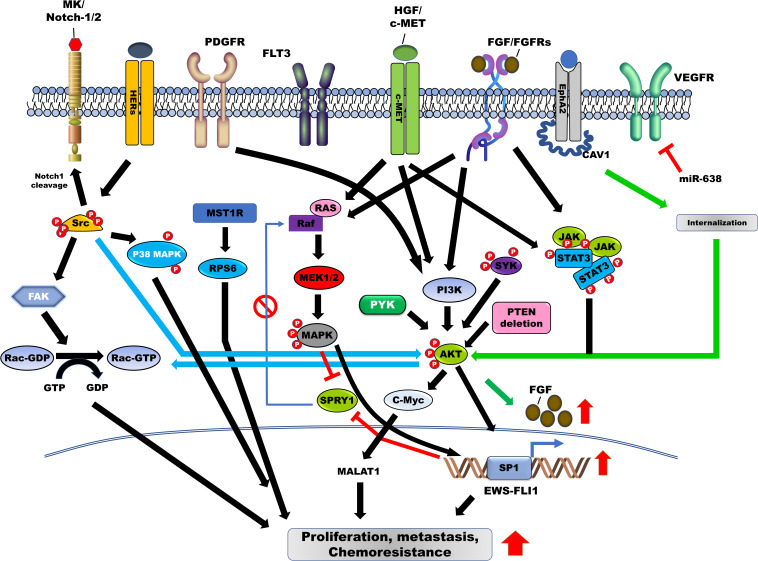
Contribution of tyrosine kinases in the induction of survival, metastasis, and chemoresistance in Ewing sarcoma (ES) through the activation of downstream signaling pathways. Tyrosine kinase receptors and non-receptor tyrosine kinase, including HGF/MET, EphA2, c-kit, HER2/3/4, ALK, PDGFR/PDGF, JAK/STAT, SFK, PYK22, FLT3, AXL, and SYK are involved in the pathogenesis of ES by blocking feedback inhibition of RAS/MAPK/ERK by SPRY1 and upregulation of EWS-FLI-1 through the activation of RAS/MAPK/ERK, PI3K/AKT, and JAK/STAT3 signaling pathways. Additionally, Src activation leads to cell proliferation and metastasis of ES cells via induction of Rac1 activity through the activation of p38 MAPK, FAK, and PI3K/AKT signaling pathways. HGF, hepatocyte growth factor; EphA2, erythropoietin-producing hepatoma receptor A2; STAT3, signal transducer and activator of transcription-3; FAK, focal adhesion kinase; MAPK, mitogen-activated protein kinase; JAK, Janus kinase.

Activation of FGFRs by their ligand, FGF, promotes tumor progression through the activation of the PI3K/AKT, RAS-MAPK, and JAK/STAT signaling pathways (Figure 2) (Babina and Turner, 2017). FGFR1 is highly expressed in ES cells, and moderate-to-high levels of FGFR1 activation were detected in 77.8% of ES patients. FGF treatment induces metastasis in ES cells by activating the PI3K/Rac1 pathway (Table 1; Kamura et al., 2010). Another report revealed that FGFR1 was upregulated in 54.3% of ES patients (Table 1; Agelopoulos et al., 2015), inducing marked EWS-FLI1 upregulation (Girnita et al., 2000a) compared with other ligands, such as insulin-like growth factor-1 (IGF-1), platelet-derived growth factor (PDGF)-BB, hepatocyte growth factor (HGF), epidermal growth factor (EGF), and TGF-β1. However, suppression of FGFR activation, by the addition of the FGF-neutralizing antibody, leads to a marked reduction in EWS-FLI-1 expression (Figure 1; Girnita et al., 2000a). Expression of SPRY1, a negative feedback inhibitor of the FGFR-activated RAS/MAPK/ERK signaling pathway, was markedly reduced by EWS-FLI1; however, its upregulated expression suppressed cell proliferation and aggressiveness of ES cells by downregulating EWS-FLI1 through the inhibition of RAS/MAPK/ERK activation (Figure 1; Cidre-Aranaz et al., 2017).

An FGFR1 mutant (FGFR1 N546K), which occurs in the tyrosine kinase domain of FGFR1 as a result of an activating mutation, has been identified in pineal tumor, peritoneal sarcoma, pilocytic astrocytoma, gastrointestinal stromal tumors (GISTs), pheochromocytomas, liposarcoma, breast sarcoma, malignant peripheral nerve sheath tumor, neuroblastoma, paraganglioma, and glioblastoma (Lew et al., 2009; Jones et al., 2013; Helsten et al., 2016; Toledo et al., 2016; Nannini et al., 2017). This mutant has also been identified in 0.02% (1/50) of ES patients, and 31.7% (23/50) of ES patients demonstrated a gain of copy number of FGFR1. FGFR1 N546K mutation promotes the constitutive activation of FRFR1 (Lew et al., 2009; Katoh, 2019), and the introduction of FGFR1 N546K in NIH3T3 cells significantly increased the proliferation and the aggressiveness of cells relative to wild-type FGFR1 (Agelopoulos et al., 2015). Furthermore, FGFR4 (FGFR4 G388A) was identified in ES and breast cancer patients, but its functional role remains unclear. Further, FGFR3 (K650E), a product of an activating mutation, was identified in glioma, non-small-cell lung carcinoma (NSCLC), ES, and liposarcoma (LPS), and correlated with poor survival in patients with LPS (Helsten et al., 2016; Li et al., 2016; Lee et al., 2018).

Table 3 shows preclinical or clinical trials using FGFR inhibitors, with or without anti-neoplastic inhibitors as targeted therapeutics against ES. Treatment with FGFR inhibitors (PD173074 [PD-74], PD166866 [PD-66], SU5402 [SU54], and NVP-BGJ398 [BG-98]) reduced the proliferation of ES cells; PD173074 in particular suppressed the proliferation of ES cells and tumor formation in mice (Cidre-Aranaz et al., 2017). Additionally, treatment with ponatinib, a known FGFR inhibitor targeting FGFR1, elicited a good response by suppressing glucose uptake in ES tumors (Agelopoulos et al., 2015).

**TABLE 3:** Efficacy of tyrosine kinase targeting inhibitors in combination with other inhibitors in treating ES.

**Target of inhibitors**	**Inhibitor/Clinical trial identifier**	**Drug Type**	**Phase**	**Efficacy of Drug**	**References**
VEGFR2	Apatinib/2017PHB176-03	Chem	Phase 1/2	62.5% of patients showed a PR, and 19.6% of patients had SD	Xie et al., 2018
VEGFR1, 2, 3	Axitinib/NCT02164838	Chem	Phase 1	31.25% of patients achieved SD, and 0.07% of patients had PR	Geller et al., 2018
VEGFR1, 2, 3 + PD-1	Axitinib + Pembrolizumab/NCT02636725	Chem + mAb	Phase 2	28% of patients achieved SD, and 25% of patients achieved PR	Wilky et al., 2019
VEGFR1, 2, 3	Pazopanib/NCT00753688	Chem	Phase 3	67% of patients achieved SD, and 6% of patients had CR	van der Graaf et al., 2012
VEGF + inhibiting DNA and protein synthesis	IFN-β + ifosfamide	cytokine + Chem	Cell line/Xg	These treatments inhibited tumor growth of ES cells	Sanceau and Wietzerbin, 2004
VEGFR2 + IGF-1R	Adnectin + AT580Peg40	Chem	Xg	This treatment effectively reduced 83.4% of tumor growth in xenografts	Ackermann et al., 2012
Glycosylation + HMG-CoA reductase	Tunicamycin + lovastatin	Chem	Cell line/Xg	These treatments reduce the activation and expression of IGF-1R and reduced the survival of ES cells	Girnita et al., 2000b
HER2	HER2-CAR-T-cell/NCT00902044	IT	Phase 1	75% of patients had a residual tumor removed, 82.3% of patients showed more than 90% prominent necrosis, and the median survival of patients markedly increased to 10.3 months	Ahmed et al., 2015
FLT3	Midostaurin	Chem	Cell line/Xg	Suppressed the survival and tumor formation of ES cells in xenograft	Boro et al., 2012
FLT3 + IGF-1R	Midostaurin + BMS-754807 or OSI-906	Chem	Cell line	This combination presented a synergistic effect on the inhibition of metastatic ability and proliferation of ES cells	Radic-Sarikas et al., 2017
MET	Cabozantinib/NCT02243605	Chem	Phase 2	49% of patients presented SD, and 22% of patients had a PR	Italiano et al., 2018
HGF + GD2	Rilotumumab + GD2-CAR-T cells	mAb	Cell line/Xg	This combination significantly reduced tumor growth and metastasis of xenografts	Charan et al., 2019
PDGFR	Linifanib	Chem	Cell line/Xg	Linifanib inhibited cell proliferation, tumor growth, and metastases of ES cells	Ikeda et al., 2010
PDGFR + topoisomerase2	Olaratumab + doxorubicin/NCT02451943	Chem	Phase 3	This treatment did not produce clinical benefits in patients with soft tissue sarcoma	U.S.F.A.D. Administration, 2019
FGFRs	PD173074	Chem	Cell line/Xg	PD173074 suppressed the proliferation of ES cells and tumor formation	Cidre-Aranaz et al., 2017
FGFRs	Rogaratinib/NCT01976741, NCT03410693, NCT03473756	Chem	Phase 1 Phase 2/3 Phase 1	Rogaratinib reduced proliferation in various cancer types but did not show any evidence of cancer reduction in ES patients in preclinical or clinical trials	UE
FGFRs	Ponatinib	Chem	Cell line	Ponatinib suppressed the proliferation of ES cells	Agelopoulos et al., 2015
FGFRs	PD166866, SU5402, NVP-BGJ398	Chem	Cell line	These inhibitors suppressed the proliferation of ES cells	Cidre-Aranaz et al., 2017
FGFRs	Erdafitinib/NCT02365597	Chem	Phase 2	2.3% of bladder patients had CR, and nearly 30% showed PR. This study is now recruiting ES patients	Montazeri and Bellmunt, 2020
AXL	Bemcentinib + vincristine	Chem	Cell line	Reduced cell viability and migration	Fleuren et al., 2014
JAK1/2	AZD1480	Chem	Cell line/Xg	AZD1480 inhibited growth and tumor formation of ES cells in xenograft	Yan et al., 2013
STAT3	LY5	Chem	Cell line/Xg	LY5 markedly decreased the cell viability of sarcomas	Yu et al., 2017

*mAb, monoclonal antibody; Chem, Chemical; SD, Stable disease; PR, Partial response; CR, Complete recovery; ORR, Objective recovery rate; UE, Under Evaluation; Xg, Xenograft; IT, Immunotherapy; VEGFR, a vascular endothelial growth factor; FGFR, fibroblast growth factor receptors; JAK, Janus kinase; STAT3, signal transducer and activator of transcription; PDGFR, Platelet-derived growth factor receptor; HGF, hepatocyte growth factor; FLT3, FMS like tyrosine kinase 3 receptor; IFN-β, interferon beta; GD, Disialoganglioside.*

In preclinical or clinical trials, rogaratinib, an ATP-competitive inhibitor of FGFR1-4, failed to demonstrate any antitumor activity in ES conducted both *in vitro* or *in vivo*. However, rogaratinib potently inhibited the kinase of FGFR1-4 in various cancers (Collin et al., 2018; Grunewald et al., 2019). These results support the inhibitory activity of rogaratinib, which is currently undergoing phase 2/3 clinical trials (ClinicalTrials.gov identifier: NCT01976741, NCT03410693, NCT03473756).

In April 2019, a clinical trial investigated FDA-approved erdafitinib, the first targeted therapy for the treatment of patients with metastatic bladder cancer with FGFR2 or FGFR3 mutations or gene fusions. Treatment with erdafitinib (ClinicalTrials.gov identifier: NCT02365597) demonstrated an ORR of 32.2%, with 2.3% of these patients eliciting a complete response and nearly 30% showing partial response (Montazeri and Bellmunt, 2020). Erdafitinib, as well as rogaratinib, exhibited antitumor activity against ES *in vitro* or *in vivo* in non-clinical studies. Clinical trials for the same are currently ongoing in patients with ES (NCT03210714).

### Human Epidermal Growth Factor Receptors and Its Inhibitors

Human epidermal growth factor receptors (HERs; ERBBs) have been identified as inducers of ES (Figure 2). HER2 (ERBB2) is overexpressed in various human cancers and is associated with poor patient survival (Moasser, 2007). Additionally, HER2 is upregulated in 16% of ES patients and 83% of ES cell lines, and this upregulation is associated with a reduced survival rate in patients. Moreover, in 78% of ES patients, HER2 expression was associated with P-glycoprotein expression (Table 1; Scotlandi et al., 2005). Furthermore, HER2 expression is associated with the tumorigenicity of ES and is overexpressed in ES cells, conferring resistance against topoisomerase II-targeting anticancer drugs, including etoposide and adriamycin. However, treatment with adenovirus early region 1A (E1A) or adenovirus-E1A therapy markedly decreased tumor formation in mice and enhanced sensitivity to etoposide and adriamycin by downregulating HER2 and upregulating topoisomerase IIα (Zhou et al., 2001; Zhou et al., 2002).

Human epidermal growth factor receptor 3 plays a crucial role in the progression of cancer as an inducer of chemoresistance, through heterodimerization with other HER receptors or through ligand binding (Baselga and Swain, 2009). In ES patients, analysis of RTK expression profiles revealed that HER3 (ERBB3) is widely expressed; however, EGFR, its heterodimer-partner, demonstrated low-level expression in ES patients. Reportedly, the expression of HER3 is associated with reduced survival in ES patients (Potratz et al., 2016).

The expression of HER4, a member of the EGFR family, is associated with ES progression. HER4 is strongly expressed in metastatic ES cells, 57.7% of primary ES patients, and 78.9% of metastatic ES patients, relative to primary ES patients. Treatment with lapatinib and a pan-ErbB inhibitor reduced the proliferation and metastatic potential of ES cells (Table 1; Mendoza-Naranjo et al., 2012; Mendoza-Naranjo et al., 2013). Moreover, HER4 enhanced the invasiveness of metastatic ES cells and increased their metastases *in vivo* by increasing Rac1 GTPase activity by activating the PI3K/AKT and FAK pathways (Mendoza-Naranjo et al., 2012, 2013). Expression of microRNA (MiR)-193b suppressed anchorage-independent growth in ES cells by downregulating HER4 as its direct target (Moore et al., 2017).

HER2 chimeric antigen receptors and lapatinib (as a HER2 and HER4 inhibitor) have been evaluated in the treatment of solid tumors, including ES. HER2-specific chimeric antigen receptor (HER2-CAR) T-cells from healthy donors markedly reduced the proliferation of HER2-positive medulloblastoma cells, and HER2-CAR-T-cells from HER2-positive patients killed primary autologous HER2-positive medulloblastoma cells *in vivo* and *in vitro* by the inducing interferon (IFN)-γ production (Ahmed et al., 2007; Thanindratarn et al., 2020). Immunotherapy with antigen-specific T-cells (HER2-CAR-T-cells) was developed and tested in patients with metastatic or recurrent sarcoma in phase I/II clinical trial (ClinicalTrials.gov identifier: NCT00902044). Reportedly, 23.5% of patients receiving HER2-CAR-T-cells demonstrated SD for 3−14 months, and 75% of the patients who had residual tumors removed reported superior outcomes. Additionally, 82.3% of patients showed more than 90% necrosis of tumor cells, and the median survival of patients increased to 10.3 months (Table 3; Ahmed et al., 2015). Recently, FDA-approved neratinib, a new HER2 inhibitor for the treatment of patients with metastatic HER2-positive breast cancer (ClinicalTrials.gov identifier: NCT01808573) (U.S.F.A.D. Administration, 2020b), decreased the risk of disease progression or death by 24% when compared with lapatinib or capecitabine, indicating that this inhibitor might provide a promising therapeutic option to improve host cytotoxicity and advance the clinical treatment of sarcoma. BDTX-189, osimertinib, and DZD9008, as novel ERBB inhibitors, are now under evaluation in phase 1/2 clinical trial in patients with solid tumors and NSCLC with mutations or alternations in HER2 or EGFR, respectively (ClinicalTrials.gov identifier: NCT04209465, NCT03586453, NCT03974022). However, these inhibitors have not been evaluated in ES.

### MET/Hepatocyte Growth Factor (HGF), Anaplastic Lymphoma Kinase (ALK)/Pleiotrophin (PTN), or ALK/Midkine (MK) and Its Inhibitors

Activation of MET by HGF, and that of ALK by PTN or MK as its ligands, renders cancer more aggressive by activating multiple downstream pathways, including the PI3/AKT and ERK pathway (Hallberg and Palmer, 2013; Duplaquet et al., 2018). MET and ALK expression are involved in the pathogenesis of ES and synovial sarcoma (Dirks et al., 2002; Fleuren et al., 2013). MET and ALK are strongly expressed in the cytoplasm of 86% ES patients, as well as in the membranes of 70% ES patients. Membranous MET expression and high ALK expression have been associated with poor survival in ES patients (Table 1; Fleuren et al., 2013). Using phosphoproteomic profiling, MET and ALK have been identified as novel targets of synovial sarcoma and are highly expressed in 58% and 14% of ES patients, respectively (Fleuren et al., 2017). In MET, missense mutations were identified in various cancer types, including ES. Patients with ES (11.1%) exhibited MET mutations such as T1010I and N375S; however, these mutations failed to demonstrate functional roles in ES (Jiang et al., 2014). Additionally, these mutations have been observed in other cancers. In breast cancer, the MET T1010I mutation increased the metastatic potential of mammary epithelial cells by induction of AKT, MAPK, and STAT3 activation. Moreover, this mutant induced tumor formation in xenografts (Figure 1; Liu et al., 2015). Furthermore, the MET N375S mutation modulates sensitivity and confers resistance to MET inhibitors, eventually suppressing MET inhibitor-mediated apoptosis in lung cancer cells (Krishnaswamy et al., 2009).

Table 3 shows preclinical and clinical trials using MET/HGF inhibitors with or without anti-neoplastic inhibitors as targeted therapeutics against ES. The FDA has approved crizotinib and cabozantinib (MET inhibitors) for patients with metastatic NSCLC, advanced renal cell carcinoma (RCC), and hepatocellular carcinoma (HCC), along with sorafenib or anti-angiogenic therapy. Crizotinib and cabozantinib have been tested in ES patients, and results indicated that they significantly reduced the viability of ES cells (Fleuren et al., 2013). Furthermore, 26% of ES patients treated with cabozantinib in a phase 2 trial demonstrated an objective response, 49% of patients showed SD, with 22% demonstrating a partial response for 6 months, thus indicating that cabozantinib possesses antitumor effects in ES patients (ClinicalTrials.gov identifier: NCT02243605) (Italiano et al., 2018).

Disialoganglioside (GD2) was upregulated in 20% of ES patients; a combination of GD2-CAR-T cells and all-*trans* retinoic acid (ATRA) significantly reduced tumor formation and prolonged survival in mice by suppressing the population of monocytic and granulocytic myeloid-derived suppressor cells (MDSCs) (Long et al., 2016). In ES cells, HGF treatment significantly increased the tumor mass and metastasis. Moreover, a combination of GD2-CAR-T cells and rilotumumab, an anti-HGF antibody (AMG102), increased mice survival by suppressing tumor growth and metastasis (Charan et al., 2019).

### Platelet-Derived Growth Factor Receptor (PDGFR)/Ligand (PDGF) and Its Inhibitors

PDGFR and its ligand (PDGF) are known therapeutic targets promoting proliferation and progression of cancer. Additionally, they are associated with the pathogenesis of ES, with PDGFRβ highly upregulated in ES cells (90.4%) and ES patients (79%) (Table 1; Uren et al., 2003). Activation of PDGFR by its ligand leads to the induction of motility in ES cells, by activation and induction of downstream targets, including PI3K/AKT and PLCγ (Figure 2; Uren et al., 2003). Additional reports revealed that the α- and β-subunits of PDGFR were strongly expressed in tissues of ES patients. High levels of PDGFRα were observed in 100% of ES patients, with high levels of PDGFRβ observed in 79% of these patients. The knockdown of PDGFRβ in ES cells markedly reduced pulmonary metastases and reduced the size of primary chest wall ES tumors in an ES orthotopic xenograft model (Table 1; Wang et al., 2009). Bone marrow (BM) cells migrate to Ewing tumors and differentiate into the vascular smooth muscle/pericyte-like cells and endothelial cells expressing α-smooth muscle actin, desmin, and PDGFRβ, as well as a vascular endothelial growth factor (VEGFR)-2, and are subsequently associated with tumor vessel endothelium by PDFGRβ to support tumor vasculature (Reddy et al., 2008).

Table 3 shows PDGFR inhibitors in combination with or without other inhibitors for the treatment of ES. Functional crosstalk between PDGF-BB/PDGFR and stromal-derived growth factor 1α (SDF-1α)/C-X-C motif chemokine receptor 4 (CXCR4) requires enhanced cancer tumorigenesis and metastasis. Autocrine activation of PDGFRα induced tumorigenesis and metastasis in advanced skin squamous cell carcinomas (SCCs) via upregulation and secretion of SDF-1α, and then SDF-1/CXCR4 induced PDGFR-induced tumor cell invasion and metastasis (Bernat-Peguera et al., 2019). Furthermore, PDGFRβ inhibitors inhibited SDF-1α-induced chemotaxis in glioblastoma (Sciaccaluga et al., 2013), and PDGF-BB induced CXCR4 and CXCR7 expression in pericytes (Xiang et al., 2019).

Plerixafor (AMD3100), a CXCR4 antagonist, inhibits the BM-mediated tumor vasculature in ES by reducing PDGFRβ expression through the disruption of the SDF-1α/CXCR4 axis (Hamdan et al., 2014). CXCR4 was markedly upregulated in 64% of therapy-naïve ES patients and 47% of metastatic ES patients. SDF-1α (CXCL12), a ligand of CXCR4, was significantly increased in 95% of therapy-naïve and metastatic ES patients, and high CXCR4 expression was correlated with reduced survival in ES patients. ES cell proliferation was markedly increased following treatment with SDF-1α; however, the addition of plerixafor suppressed the proliferation of ES cells (Berghuis et al., 2012).

The efficacy of PDGFR inhibitors against ES has been evaluated. Linifanib (ABT-869) is a multitargeting tyrosine kinase inhibitor, which targets tyrosine kinases such as VEGFR, FLT3, and PDGFR. Linifanib is clinically active in patients with HCC, acute myeloid leukemia (AML), renal cell cancer, and NSCLC (Lowery et al., 2018), inhibiting cell proliferation, tumor growth, and metastases in ES cells in xenografts by suppressing PDGFRβ and c-kit activation (Ikeda et al., 2010). PDGFRβ signaling-mediated pericyte recruitment facilitated the formation of the vascular-like structure by ES cells. However, treatment with imatinib- or a PDGFR-blocking antibody inhibited pericyte recruitment by suppressing PDGFRβ signaling, subsequently reducing tumor growth in ES cells *in vivo* (Thijssen et al., 2018).

Olaratumab, a PDGFRα inhibitor, delayed tumor formation in pediatric bone and soft tissue tumors in mice (Lowery et al., 2018); however, a combination of olaratumab and doxorubicin (ClinicalTrials.gov identifier: NCT02451943) failed to demonstrate clinical benefits in patients with soft tissue sarcoma (U.S.F.A.D. Administration, 2019). Instead, FDA-approved avapritinib (Ayvakit), another PDGFRα inhibitor, was investigated in clinical trials to treat patients with gastrointestinal stromal tumors (GISTs) presenting a PDGFRA D842V mutation, demonstrating a high response rate in 85% of patients. To date, no such clinical trials or preclinical examinations have been conducted in ES patients.

### FMS Like Tyrosine Kinase 3 Receptor (*FLT3*; *CD135*) and Its Inhibitors

FMS like tyrosine kinase 3 (*FLT3*; *CD135*) is associated with poor clinical outcome in AML as it promotes cell proliferation and survival through the activation of the PI3K, RAS, and STAT3 signaling pathways (Stirewalt and Radich, 2003), and is reportedly mutated in approximately 30% of AML patients (Figure 2; Daver et al., 2019). These mutations lead to ligand-independent activation of FLT3 and its downstream pathway (Stirewalt and Radich, 2003). The expression of FLT3 and its ligand was markedly upregulated in 89.4% of ES patients (Table 1; Timeus et al., 2001).

Table 3 shows the FLT3 inhibitors investigated for the treatment of ES. Midostaurin (PKC412), an FLT3 inhibitor, first approved by the FDA for treatment of AML patients with FLT3 mutations, suppresses the survival of ES cells and tumor growth in mice (Boro et al., 2012). A combination of midostaurin and IGF-1R/INSR inhibitors (BMS-754807 and OSI-906), identified through specific drug-drug interactions by a parallel combinatorial drug screen, has demonstrated a synergistic effect by inhibiting the metastatic ability and proliferation of cells through the suppression of PKC/AKT/mTOR and MAPK signaling pathways (Radic-Sarikas et al., 2017). Recently, gilteritinib (XOSPATA), a novel FLT3 inhibitor, was approved by the FDA for relapsed or refractory AML patients with FLT mutation (ClinicalTrials.gov identifier: NCT0241939). In AML patients receiving gilteritinib, the OS (9.3 vs. 5.6 months) and EFS (2.8 vs. 0.7 months) rates were significantly increased relative to the salvage chemotherapy group. Moreover, the number of patients with complete remission, with full or partial hematologic recovery, was 2.2 fold higher when compared with the salvage chemotherapy group (Perl et al., 2019). Treatment with gilteritinib may contribute to the better survival of ES patients, although its effects have not been investigated in ES patients.

### Interferon-α/β Receptor (IFNAR)/Interferon-β (IFN-β) and IFN-β Based Therapy

Treatment with IFN-β induces activation of p38 mitogen-activated protein kinase (MAPK) by increasing serine phosphorylation of STAT1, inducing apoptosis in ES cells by activating caspase-7 through the induction of interferon response factor (IRF)-1, thereby inducing cell cycle arrest in cooperation with p53 through transactivation of p21^*waf*1/CIP1^ (Sanceau et al., 2000).

As the FDA has approved IFN-based therapies for multiple sclerosis (English and Aloi, 2015) in the U.S., treatment with IFN-β (Betaseron, Extavia), which has direct and indirect effects on tumor inhibition, will soon be utilized as a new therapeutic strategy for cancer (Borden, 2019). IFN-β demonstrates potential anti-tumorigenic effects on tumor cells, as well as immune-infiltrating cells (Musella et al., 2017), reducing tumor cell growth in various type of cancers through by suppressing PI3K/AKT activation, reducing anti-apoptotic proteins, including FLICE (FADD-like IL-1β-converting enzyme)-inhibitory protein (c-FLIP) and caspase-8, and activating the TNF-related-apoptosis-inducing ligand (TRAIL)-dependent pathway (Chawla-Sarkar et al., 2001; Dedoni et al., 2010; Apelbaum et al., 2013). Moreover, IFN-β repressed the acquisition of CSC characteristics (Doherty and Jackson, 2018); treatment with IFN-β markedly suppressed tumor growth in ES cells (Table 3). Furthermore, IFN-β treatment combined with ifosfamide demonstrated a strong antitumor effect by potently inhibiting tumor growth in ES cells in a xenograft model (Sanceau et al., 2002), by drastically reducing the expression of angiogenesis mediators, including VEGF, matrix metalloproteinase (MMP)-9, and urokinase plasminogen activator receptor (uPAR) (Sanceau and Wietzerbin, 2004).

### Vascular Endothelial Cell Growth Factor A (VEGFA)/VEGF and Its Inhibitors

Vascular Endothelial Cell Growth Factor A and its ligand VEGF are involved in the pathogenesis of ES. VEGF isoform (VEGF_165_) expression significantly increased cell proliferation and tumor growth in ES cells by stimulating the migration of BM to the tumor, by increasing tumor vessel density (Lee et al., 2006). Furthermore, miR-638, which inhibits cell proliferation by targeting Sox-2, suppressed the expression of VEGFA in ES cells through direct targeting (Figure 2; Ma et al., 2014; Zhou et al., 2018). Moreover, the secretion and expression of VEGFA in ES cells were induced by IGF-mediated activation of PI3K/AKT and MAPK signaling pathways (Figure 1; Strammiello et al., 2003).

Additionally, VEGFR inhibitors were evaluated as vascular targeting agents for the treatment of ES (Table 3). Apatinib (rivoceranib), a novel VEGFR2 inhibitor, showed stabilized disease symptoms in 19.6% sarcoma patients and a partial response in 62.5% patients. The ORR with apatinib was 70% for ES, 40.9% for osteosarcoma, 100% for chondrosarcoma, and 71.4% for soft tissue sarcoma (Xie et al., 2018), indicating that combination therapy with IGF-1R may be therapeutically more efficient for cancer patients.

Following the administration of axitinib, another VEGFR 1–3 inhibitor, 31.25% of recurrent or refractory solid tumor patients, including ES, achieved SD, and 0.07% of patients showed partial response (ClinicalTrials.gov identifier: NCT02164838) (Geller et al., 2018). Additionally, axitinib in combination with pembrolizumab, an anti-programmed cell death protein 1 (PD-1) monoclonal antibody for advanced sarcoma patients, demonstrated that 28% of patients achieved SD and 25% showed partial response (ClinicalTrials.gov, number NCT02636725) (Wilky et al., 2019).

Pazopanib is an inhibitor of multiple protein tyrosine kinases, including VEGFR 1-3, c-kit, and PDGFR. In phase 3 clinical trial (ClinicalTrials.gov, number: NCT00753688), treatment with pazopanib, a synthetic indazolpyrimidine, revealed a 2.87-fold higher PFS in sarcoma patients when compared with the placebo group. Pazopanib treatment resulted in SD in 67% of patients, demonstrating a partial response in 6% of patients (van der Graaf et al., 2012). In metastatic ES patients, the administration of pazopanib produced significant responsiveness with complete tumor regression and prolonged survival for more than 26 months (Mori et al., 2018). Moreover, pazopanib maintenance therapy in ES patients, following high-dose chemotherapy with topotecan, significantly prolonged PFS, with near-complete remission for 1 year (Tamura et al., 2019).

Adnectin, a VEGFR2 inhibitor, in combination with AT580Peg40, an IGF-1R inhibitor, reduced tumor growth by 83.4% in mice by normalizing the microvascular architecture via inhibition of autocrine VEGF-secretion and downregulation of IGF-1R. Moreover, AT580Peg40 and adnectin monotherapies yielded 37.18% and 73.39% tumor growth inhibition, respectively (Ackermann et al., 2012). These monotherapies or a combination therapy presented manageable toxicities; therefore, such therapies can be promising new treatment options.

### AXL Receptor Tyrosine Kinase/Gas6 and Its Inhibitors

AXL receptor tyrosine kinase, together with its ligand, growth-arrest-specific protein 6 (Gas6), is a new therapeutic target overexpressed in various cancers and significantly associated with tumor proliferation, metastasis, EMT, and acquisition of CSC characteristics (Gay et al., 2017; Zhu et al., 2019). Moreover, AXL expression is associated with the pathogenesis of ES. Medium and high levels of AXL were detected in 69% of ES patients (Table 1). High AXL expression worsened the survival rate in ES patients. Bemcentinib (BGB324), an AXL inhibitor, in combination with vincristine (a mitotic inhibitor), showed a synergistic therapeutic effect, reducing cell viability and migration of high AXL- and Gas6-expressing ES cells (Table 3; Fleuren et al., 2014).

### Involvement of Erythropoietin-Producing Hepatoma (EPH), Receptor A2 (EphA2), and c-kit (CD117) Stem Cell Receptor in the Pathogenesis of ES

The activation of the erythropoietin-producing hepatoma (EPH) receptor A2 (EphA2) is involved in FGF production. EphA2 is highly expressed in ES cells and ES patients. CAV1, an activator of EphA2, increased EphA2 activation through proper localization of EphA2, via the formation of the CAV1/EphA2 complex. CAV1-mediated EphA2 activation suppressed cell death and impaired vascularization in ES xenograft models by increasing the migration of endothelial cells and inducing the expression and secretion of FGF through AKT activation (Figure 2; Sainz-Jaspeado et al., 2013). Phosphorylation at S897 (p-EphA2^*S*897^) of EphA2 correlated with the aggressiveness of ES. High p-EphA2^*S*897^ levels significantly increased the proliferation and migration of ES cells when compared with low phosphorylation levels; however, its mutant markedly suppressed the metastatic potential of ES cells. EphA2 expression was observed in 90.4% of ES patients, and this phosphorylation was associated with a lower survival rate in ES patients. Additionally, EphA2 demonstrated a strong correlation with cellular movement and survival-related signaling, such as the EGFR signaling pathway (Table 1; Garcia-Monclus et al., 2018).

c-kit (CD117), a tyrosine kinase receptor, plays a crucial role in the development of melanogenesis, hematopoiesis, and gonadogenesis (Nocka et al., 1989), and is highly expressed in soft tissue sarcoma cells of neuroectodermic origin, such as ES and peripheral neuroectodermal tumors (PNET). Activation of c-kit by its ligand stem cell factor (SCF) subsequently induces cell proliferation (Ricotti et al., 1998). Previously, two separate reports revealed that c-kit was significantly upregulated in 65% of ES patients and 38% of ES patients, respectively (Table 1; Ahmed et al., 2004; Do et al., 2007). Another report documented that 71% of ES and 100% of synovial sarcoma patients were positive for c-kit; additionally, 29 and 64% of ES and synovial sarcoma patients showed strong and diffuse staining for c-kit, respectively (Table 1; Smithey et al., 2002). However, treatment with soluble SCF, presenting biological activities distinct from membrane-bound SCF, markedly decreased cell migration, metastasis to the lungs and extrapulmonary sites, and death due to metastasis in mice (Landuzzi et al., 2000).

Imatinib inhibits tumor growth of ES cells *in vivo* and *in vitro* by suppressing activation of SCF-mediated c-kit (Merchant et al., 2002); however, a clinical trial evaluating imatinib failed to demonstrate clinical benefits in patients with ES (ClinicalTrials.gov Identifier: NCT00154388) (Chugh et al., 2009). In another study, 14.3% of ES patients exhibited partial response without progression (ClinicalTrials.gov Identifier: NCT00062205) (Chao et al., 2010). Regorafenib treatment, as a multikinase inhibitor, including c-Kit, VEGFR1-3, BRAF, FGFR1, significantly increased the EFS in 100% (9/9) of sarcoma models, including osteosarcoma, rhabdomyosarcoma, and ES (Harrison et al., 2019), and is currently under evaluation in phase 2 clinical trial for patients with sarcoma subtypes, including ES (ClinicalTrials.gov identifier: NCT02048371).

## The Functional Role of Non-Receptor Tyrosine Kinases in ES Progression and on Its Inhibitors

### The Janus Kinase (JAK)/Signal Transducer and Activator of Transcription (STAT) and Its Inhibitors

The JAK/STAT pathway plays a major role in the progression of various types of cancers (Jones and Jenkins, 2018), including ES. STAT3 activation was identified in 51% of ES patients and primary ES cells. However, STAT3 activation failed to correlate with EWS-FLI1 (Lai et al., 2006). Another report demonstrated that STAT3 was activated in 58% of ES patients and ES cells (Behjati et al., 2012). The activation of STAT3 was markedly increased by a W775 stop germline mutation of protein tyrosine phosphatase delta, a tumor suppressor, identified in 37.5% of metastatic ES patients (Veeriah et al., 2009; Jiang et al., 2013; Jiang et al., 2014). Treatment with epigallocatechin gallate, major catechin present in green tea, inhibited proliferation, survival, and invasiveness of ES cells by suppressing its downstream pathways, including PI3/AKT, JAK2/STAT3, and by RAS/ERK signaling through the inhibition IGF-1R activation (Kang et al., 2010).

The secretion of cytokines enhances the progression of ES. Oncostatin M (OSM), a cytokine of the interleukin-6 (IL-6) family, as well as its receptors, induced cell proliferation in ES cells by activation of STAT3 and Myc (David et al., 2012). Furthermore, the high levels of IL-6, secreted by ES-stroma crosstalk in ES tumor microenvironment, activated the JAK/STAT3 signaling and inducing resistance to the IGF-1R blockade; however, the combination of IGF-1R and STAT3 inhibitors significantly increased sensitivity against the resistance to IGF-1R (Lee et al., 2015; Santoro et al., 2017).

Janus Kinase/STAT inhibitors have been investigated as monotherapy for the treatment of ES (Table 3). ViscumTT treatment, a whole mistletoe extract, significantly reduced tumor growth in ES cells *in vivo* through the JAK/STAT3 pathway and its downstream targets, including BIRC5 and Myc (Twardziok et al., 2016; Kleinsimon et al., 2018). AZD1480, a JAK1/2 inhibitor, exhibited antitumor activity by inhibiting the growth of ES cells and tumor formation in xenograft models via the suppression of the IL-6/JAK2/STAT3 signaling pathway. Furthermore, AZD1480 treatment significantly increased the survival of ES tumor-bearing xenograft mice (Yan et al., 2013). Another small molecule inhibitor, LY5, which inhibits homodimerization by blocking the phosphotyrosine site of the STAT3 SH2 domain, inhibited STAT3 activation by decreasing the cell viability of sarcomas, including osteosarcoma, ES, and RMS. LY5 treatment showed excellent oral bioavailability in both mice and dogs, with 69.5% bioavailability observed following intraperitoneal administration and 78.6% with oral administration. Although treatment with LY5 inhibits the activation of STAT3 in metastatic pulmonary lesions of sarcoma xenografts, it failed to suppress lung metastasis *in vivo* (Yu et al., 2017).

### Src Family Tyrosine Kinase (SFK) and Its Inhibitors

In cancer pathogenesis, SFK is essential for growth and metastasis (Kim et al., 2009) and its roles have been well established in cancer, with several studies reporting the functional role of SFK in the pathogenesis of ES. Although Src is highly expressed and activated in ES cells and ES patients, its expression demonstrated no significant correlation with histologic subtypes. Dasatinib, an Src inhibitor, induced apoptosis and reduced the invasiveness of ES cells by suppressing the downstream signaling pathways, such as FAK and p130^*CAS*^, via inhibition of Src activity (Figure 1; Shor et al., 2007). Using ES cells, phosphoproteomics has shown that SFK is an abundant phosphorylated tyrosine kinase, and dasatinib treatment markedly reduced cell viability in ES cells by inhibiting the activation of PDGFRα and Src (Bai et al., 2012). Furthermore, peptide-based tyrosine kinase array revealed that Src is highly expressed and phosphorylated in *CIC-DUX4* sarcoma (CDS) patient-derived xenografts, which is a high-grade Ewing-like small round cell sarcoma (Oyama et al., 2017). Lyn, a member of SFK, was upregulated and activated in 86.7% of ES patients and ES cells (Table 1). However, the downregulation of SFK and treatment with AP23994, an SFK inhibitor, suppressed tumor growth, bone tumor lysis, and metastases in ES cells and the xenograft model (Guan et al., 2008). Microenvironmental stress, including growth constraints, serum deprivation, and hypoxia, induced the invasiveness of ES cells through the formation of invadopodia by Src activation, not through the induction of EMT markers and cell proliferation (Bailey et al., 2016).

Secretion of the extracellular matrix protein, tenascin C, upon microenvironmental stress, induced cell-cell interaction, and growth of ES cells by promoting invadopodia formation by Src activation. In contrast, dasatinib treatment suppressed the environmental stress-mediated invasiveness of ES cells by blocking invadopodia formation associated with Src activation and tenascin (Bailey et al., 2016; Hawkins et al., 2019). SI221, a new pyrazolo[3,4-*d*]pyrimidine SFK inhibitor, reduced cell proliferation and migration of ES cells by inhibiting NOTCH1 cleavage and activating P38 MAPK (Indovina et al., 2017). Additionally, dasatinib has been evaluated in phase 2 clinical trial in sarcoma patients (ClinicalTrials.gov Identifier: NCT00464620), revealing that the 6 month PFS rate was 48%, and more than 50% of patients with alveolar soft part sarcoma (ASPS) and epithelioid sarcoma showed disease-free progression for more than 6 months (Schuetze et al., 2017). Furthermore, dasatinib, in combination with other conventional agents (ifosfamide, carboplatin, and etoposide), ids currently under investigation in phases 1 and 2 trials for patients with solid tumors, including sarcoma (ClinicalTrials.gov Identifier: NCT00788125).

### Focal Adhesion Kinase (FAK) and Its Inhibitors

Focal adhesion kinase has been identified as a therapeutic target for cancer, inducing tumor growth, metastasis, acquisition of CSC traits, and drug resistance (Sulzmaier et al., 2014; Yoon et al., 2015). FAK is significantly upregulated in ES cells (Moritake et al., 2003) and highly activated in ES patients and ES cells. Loss of FAK and treatment with a FAK inhibitor (PF-562271) suppressed tumor growth and the invasion ability of ES cells *in vivo* and *in vitro* by suppressing Crkl-associated substrate (CAS) activity and the AKT/mTOR pathway (Crompton et al., 2013). EWS-FLI1-mediated upregulation of ezrin enhanced the viability and invasion of ES cells via FAK activation (Steinestel et al., 2020). Furthermore, the upregulation of ERBB4 in metastatic ES patients was closely associated with poor survival in ES patients, and ERBB4-mediated activation of the PI3K/AKT/FAK/RAC1 pathway promoted the metastatic capacity of ES cells (Mendoza-Naranjo et al., 2013).

Moreover, rapamycin treatment or loss of TORC1 and TORC2, as mTOR complexes, markedly reduced IGF-1-mediated activation of FAK in ES cells (Liu et al., 2008). Furthermore, aurora kinase B (AURKB) inhibitors have been identified as a synergistic drug in combination with FAK inhibitors by utilizing high-throughput drug combination screening, with AURKB highly expressed in ES patients and ES cells. AURKB inhibitor significantly suppressed cell viability, and the combination of the FAK and AURKB inhibitors demonstrated synergistic activity by inhibiting activation of the mTOR pathway (Wang et al., 2019).

Clinical activity of GSK2256098, a FAK inhibitor, has been evaluated in cancer patients. GSK2256098 monotherapy was well-tolerated in patients with relapsed glioblastoma, and SD was observed in 27% (3/11) of patients (ClinicalTrials.gov: NCT01138033) (Brown et al., 2018). In patients with mesothelioma or solid tumors, combination treatment with GSK2256098 and trametinib, a MEK inhibitor, demonstrated that 38% (13/34) of patients presented SD and 50% (3/6) patients demonstrated more than a 70% decrease in phosphorylated FAK/FAK (ClinicalTrials.gov Identifier: NCT01938443) (Mak et al., 2019). Another FAK inhibitor, defatinib (VS-6063), was well-tolerated to patients with solid tumors, with 33% (3/9) patients presenting SD (Shimizu et al., 2016). The combination of defatinib with RO5126766 (RAF/MEK inhibitor) or pembrolizumab (PD-1 blocking antibody) is currently under evaluation for patients with solid tumors (ClinicalTrials.gov Identifier: NCT03875820, NCT02546531, NCT02758587), but remains to be evaluated in ES patients.

### Other Non-receptor Tyrosine Kinases in the Pathogenesis of ES

Activation of ERK 1 and 2 by EWS/FLI-1 induces the transformation of cells. Moreover, a dominant-negative RAS and EWS/FLI-1 mutant, defective in DNA-binding and transcriptional activation, significantly decreased activation of ERK1/2 and transformation of cells (Silvany et al., 2000).

The C-terminal portion of proline-rich tyrosine kinase (PYK2), involved in the pathogenesis of many cancers, interacts with the N-terminal region (1-233 amino acid) of EWS and eventually regulates G-protein-coupled receptor signaling (Felsch et al., 1999). Additionally, suppression of PYK2 activity inhibited the AKT/mTOR pathway (Crompton et al., 2013).

Previous studies have demonstrated that activation of spleen tyrosine kinase (SYK) increases the risk of chemotherapy failure or relapse of B cell acute lymphoblastic leukemia (B-ALL) (Perova et al., 2014), and its inhibitors, PRT318 and P505-15, inhibited the survival and migration of chronic lymphocytic leukemia (CLL) by blocking the activation of chemokine receptors (CXCR4, CXCR5) and SYK (Hoellenriegel et al., 2012). SYK was identified as a clinical target through a high-throughput approach using a siRNA library and a small-molecule library, and was reportedly activated in 40% of ES patients (Table 1). SYK enhanced cell survival and tumor growth in xenografts. Additionally, MALAT1, a non-coding RNA, transcriptionally activated through SYK/c-MYC pathway, enhanced the malignant traits of ES cells; however, its inhibitors suppressed cell viability and tumor formation in ES cells *in vivo* and *in vitro* via the inhibition of the AKT pathway (Figure 1; Sun et al., 2017).

## Conclusion

Recent studies have demonstrated that activation of several tyrosine kinases, identified as potential therapeutic targets, enhances tumor growth and metastasis, developing chemoresistance in ES cells through the activation of various intracellular downstream signaling pathways. Moreover, drugs targeting specific tyrosine kinases have been developed and approved. Although clinical observations and preclinical trials have reported that monotherapy, as well as combination therapy with such inhibitors, can efficiently combat ES, further investigations with novel multifaceted therapeutic strategies are required. IGF-1R directed therapies have demonstrated only modest efficacy in ES patients; monotherapy and combination therapy with tyrosine kinase inhibitors remain under evaluation. Additionally, the inhibition of kinase activity in ES reduced the sensitivity to the tyrosine kinase inhibitor by developing resistance. Cancer cells acquire drug resistance via mutations that abrogate inhibitor binding. These mutations upregulate oncogenes and activate alternative kinase pathways via compensatory cross-signaling through the tyrosine kinase-induced activation of target networks. The subsequent development of therapeutic resistance to cancer increases the evasion of tyrosine kinase inhibitors (Zhang et al., 2009). Additional mutations on resistant mutants, to inhibit the developed drug resistance, can increase their sensitivity to cancer cells and prolong patient survival (Ferguson and Gray, 2018). Several strategies have been proposed to overcome drug-resistant mutations by developing inhibitors such as nilotinib and dasatinib, which possess increased affinity to the ATP binding site and can recognize active conformations of tyrosine kinase. These two compounds have high cellular activity and inhibit all known BCR-ABL1 mutants (Weisberg et al., 2005; Karaman et al., 2008; Zhang et al., 2009; Ferguson and Gray, 2018).

Adoptive cell therapy and a combination of tyrosine kinase targeting agents, along with other conventional agents, may provide novel options to cancer patients to overcome drug resistance and optimize their efficacy and cytotoxicity. The FDA has approved axicabtagene ciloleucel and tisagenlecleucel as CD19 targeting CAR-T cell immunotherapy in patients with B cell lymphoma and acute lymphoblastic leukemia; these agents have shown a safe and improved clinical outcome in the treatment of cancer patients (Depil et al., 2020). Currently, CAT-T cell immunotherapy is under evaluation in clinical trials for ES patients presenting sarcoma-specific surface antigens targeting next-generation CAR-T (NCT03356782), GD2-CAR-T (NCT03635632), and EGFR806-CAR-T immunotherapy (NCT03618381). Additionally, histone deacetylase (HDAC) inhibitors, in combination with other conventional agents, have exhibited anticancer effects in preclinical and clinical studies (Suraweera et al., 2018). Vorinostat, in combination with vincristine, irinotecan, and temozolomide, is now under evaluation for ES patients (ClinicalTrials.gov Identifier: NCT04308330). Moreover, significant advances in understanding the functioning of tyrosine kinases in cancer, including identification of molecular mechanisms and the association between tyrosine kinases and the pathogenesis of ES will provide new insights for the selection of novel targets and to further overcome challenges associated with existing treatment strategies for ES. Furthermore, understanding the tumor-initiating abilities of tyrosine kinases and developing strategies to maintain this crucial property will increase success rates and improve outcomes in cancer therapy.

## Author Contributions

WJ designed, reviewed, and wrote the manuscript.

## Conflict of Interest

The author declares that the research was conducted in the absence of any commercial or financial relationships that could be construed as a potential conflict of interest.
